# Brain selective transgene expression in zebrafish using an NRSE derived motif

**DOI:** 10.3389/fncir.2012.00110

**Published:** 2012-12-28

**Authors:** Sadie A. Bergeron, Markus C. Hannan, Hiba Codore, Kandice Fero, Grace H. Li, Zachary Moak, Tohei Yokogawa, Harold A. Burgess

**Affiliations:** Program in Genomics of Differentiation, Unit on Behavioral Neurogenetics, Eunice Kennedy Shriver National Institute of Child Health and Human DevelopmentBethesda, MD, USA

**Keywords:** transgenesis, enhancer trap, ablation, NRSE, rest, zebrafish

## Abstract

Transgenic technologies enable the manipulation and observation of circuits controlling behavior by permitting expression of genetically encoded reporter genes in neurons. Frequently though, neuronal expression is accompanied by transgene expression in non-neuronal tissues, which may preclude key experimental manipulations, including assessment of the contribution of neurons to behavior by ablation. To better restrict transgene expression to the nervous system in zebrafish larvae, we have used DNA sequences derived from the neuron-restrictive silencing element (NRSE). We find that one such sequence, REx2, when used in conjunction with several basal promoters, robustly suppresses transgene expression in non-neuronal tissues. Both in transient transgenic experiments and in stable enhancer trap lines, suppression is achieved without compromising expression within the nervous system. Furthermore, in REx2 enhancer trap lines non-neuronal expression can be de-repressed by knocking down expression of the NRSE binding protein RE1-silencing transcription factor (Rest). In one line, we show that the resulting pattern of reporter gene expression coincides with that of the adjacent endogenous gene, *hapln3*. We demonstrate that three common basal promoters are susceptible to the effects of the REx2 element, suggesting that this method may be useful for confining expression from many other promoters to the nervous system. This technique enables neural specific targeting of reporter genes and thus will facilitate the use of transgenic methods to manipulate circuit function in freely behaving larvae.

## Introduction

Random mutagenesis screens have provided profound insights into neuronal circuits that control behavior in simple invertebrate species (Benzer, 1971; Brenner, 1974). Such screens have proven to be a less efficient tool for investigating the neuronal basis of behavior in vertebrates, as mutations that alter behavior often disrupt brain architecture so broadly that it is difficult to link behavioral phenotypes to specific structural deficits, or result in changes to circuit function so subtle that they are difficult to recognize. An appealing alternative is to generate transgenic lines with unique patterns of labeled neurons by exploiting *cis* regulatory DNA elements involved in the regulation of gene expression (Pfeiffer et al., 2008). Using a large collection of such lines to drive the expression of transgenes whose products interfere with neuronal function, a “circuit breaking” screen can be performed, testing for behavioral deficits which result from inactivation of the targeted neurons (Asakawa et al., 2008). Such an approach retains the unbiased nature of mutagenesis screening but disrupts brain function at a level suitable for circuit analysis.

Several methods have been developed for conditional inactivation of neurons. Conditional silencing technologies have the advantage of reversibility (Kitamoto, 2001; Tan et al., 2006; Zhang et al., 2007; Chow et al., 2010), however, proving that neurons are silenced requires electrophysiological characterization, making this technology best suited for testing specific hypotheses rather than for conducting behavioral screens. In contrast, neuronal ablation using genetically encoded toxins can be quickly monitored using co-expressed fluorescent reporters or histological techniques (Nirenberg and Meister, 1997; Chu et al., 2008; Hamm et al., 2009; Del Bene et al., 2010; Lee et al., 2010). It has recently been shown that the bacterial gene *nfsB*, which encodes the enzyme nitroreductase (NTR), can be used for conditional ablation of cells including neurons in zebrafish. NTR converts the cell-permeable substrate metronidazole into a cell-impermeable cytotoxin preventing damage to neighboring cells (Curado et al., 2007; Pisharath et al., 2007; Zhao et al., 2009; Fernandes et al., 2012). Together with advances in transgenic technology that allow for rapidly generating libraries of zebrafish enhancer trap lines (Kawakami et al., 2000; Kawakami, 2004; Emelyanov et al., 2006; Urasaki et al., 2006; Koga et al., 2008), this opens the possibility of screening for neuronal function by using these lines to drive expression of a temporally controlled cell death gene.

Enhancer trap screens have generated numerous stable transgenic lines with reporter genes expressed in restricted neuronal patterns (Parinov et al., 2004; Ellingsen et al., 2005; Davison et al., 2007; Scott et al., 2007; Asakawa et al., 2008; Distel et al., 2009; Scott and Baier, 2009), but most such lines express transgenes not only in the brain, but also in non-neuronal tissues including heart, muscle, skin, and notochord (Scott et al., 2007). Ablation of these structures either kills or produces morphological abnormalities in larvae which preclude behavioral testing. To surmount this difficulty we sought to develop a method for generating transgenic lines that would permit selective ablation of neuronal structures.

The RE1-silencing transcription factor (Rest) recognizes a 21 bp DNA motif, the neuron-restrictive silencing element (NRSE) and represses gene expression by recruiting a complex of silencing factors (Mori et al., 1990; Kraner et al., 1992; Mori et al., 1992; Andres et al., 1999; Huang et al., 1999; Ding et al., 2009). The NRSE is found mainly in the regulatory regions of neuronal genes, whereas Rest is primarily expressed in non-neuronal tissues and neural progenitor cells, acting to silence premature and non-specific neuronal gene expression (Chong et al., 1995; Schoenherr and Anderson, 1995; Chen et al., 1998). NRSE sites are abundant in vertebrates including zebrafish (Mortazavi et al., 2006) and the zebrafish *rest* gene is expressed in a pattern consistent with a role in silencing neuronal gene expression in immature neuronal progenitor cells and outside the nervous system (Gates et al., 2010).

We hypothesized that adding a NRSE to the transgene promoter would suppress reporter expression in non-neuronal tissues. Here we define a short DNA sequence based on a combination of two NRSE motifs that can be easily incorporated into existing enhancer trap vectors that improves the recovery of brain specific enhancer trap lines 5-fold in 4 days post fertilization (dpf) larvae. This opens the possibility of combining enhancer trapping with genetic ablation to screen for elements of neuronal circuits controlling behavior.

## Materials and methods

### Zebrafish lines

Tuebingen long fin strain zebrafish were used in this study. Embryos were raised in E3 medium supplemented with 1.5 mM HEPES pH 7.3 (E3h) at 28°C on a 14 h:10 h light:dark cycle with medium changes every 2 days. Transgenic lines were generated using *Tol2* mediated transgenesis (Kawakami, 2004). For transient transgenic experiments in *Tg(UAS:Kaede)s1999t* embryos (Davison et al., 2007), we used the offspring of homozygous transgenic fish. To identify homozygotes, we first used linker-mediated PCR (see below) to locate the transgene insertion site to chr5:32007986 (zv9). Genomic PCR using primers GenKL 5-tgcgtagaaaatagctttgga and TraKR 5-cttggaggcctaagcttgat amplify a 247 bp band from the transgene containing allele, and primers GenKL and GenKR 5-ccatttgttggtttgcattt amplify a 374 bp band from the wildtype genomic allele. To generate low frequency mosaic reporter expression (Downes et al., 2002), we injected 5 pg linearized plasmid (35 pg for REx4-SCP1:Gal4 which strongly suppressed expression) without transposase and raised embryos in 0.003% phenylthiourea (Sigma) until 48 hpf. The amount of plasmid was calibrated to produce only a few cells expressing the reporter per embryo, with around 50% of embryos showing no expression in the tissues scored. Embryos were inspected for the presence of any cells expressing Kaede in brain, heart, muscle, notochord, and skin. All *in vivo* experimental protocols were approved by the local animal care and use committee.

### Plasmid construction

To make Gal4 reporter constructs, Gal4ff (a variant of Gal4 engineered to reduce toxicity) (Asakawa et al., 2008) was subcloned from pT2KhspGFF into plasmid pBT2 which contains Tol2 arms (gift of Shannon Fisher), with the sequence modified to create a multiple cloning site and a canonical GCCACC Kozak sequence before the ATG (Distel et al., 2009), creating plasmid pT2MCSkG4FF. To make cFos:kGal4ff, we inserted an oligonucleotide encoding the basal cFos promoter (5-CCAGTGACGTAGGAAGTCCATCCATTCACAGCGCTTCTATAAAGGCGCCAGCTGAGGCGCCTACTACTCCAACCGCGACTGCAGCGAGCAACT) (Dorsky et al., 2002; Fisher et al., 2006; Scott and Baier, 2009) between SalI and NcoI sites in pT2MCSkG4FF. For SCP1:kGal4ff, we inserted an oligonucleotide encoding the super core promoter 1 (5-GTACTTATATAAGGGGGTGGGGGCGCGTTCGTCCTCAGTCGCGATCGAACACTCGAGCCGAGCAGACGTGCCTACGGACCG) between SalI and BamHI sites in pT2MCSkG4FF. The cFos:kGal4ff and SCP1:kGal4ff vectors were subsequently each linearized upstream of the basal promoter with EcoRV, and ligated to an oligonucleotide containing the REx2 element (REx2, NRSE elements underlined: 5-TCAGCACCACGGACAGGAAGATTTACCATACCGACAATTACTATCAGCACCGCGGACAG). The 16 bp NRSE elements used were computationally identified as the core of common *Fugu* NRSE sequences (Bruce et al., 2004). For pilot experiments with SCP1:kGal4ff, we also tested a single NRSE element (REx1: 5-TCAGCACCACGGACAG), two different NRSE elements with a 27 bp spacer (REx2), or tandem copies of the REx2 element (REx4). For testing the REx2 with the heat shock promoter (Halloran et al., 2000), we inserted an oligonucleotide containing the REx2 sequence into the ApaI site of pT2KhspGFF (Asakawa et al., 2008).

### *In situ* hybridization and immunohistochemistry

Larvae were fixed in 4% paraformaldehyde. Standard colorimetric whole-mount *in situ hybridization* (WISH) and immunohistochemistry was performed as described in Bergeron et al. (2008). For WISH, a *hapln3* fragment was amplified by RTPCR from 24 hpf embryonic RNA (primers 5-GATGGTCTGGAGGACGAGAG and 5-TGTCCCATTCCACAGAAGTG) and cloned into pGEM-T Easy (Promega, Madison, WI). Antibodies were anti-Kaede (PM012, 1:200, MBL International, Woburn, MA) and anti-elavl (16A11, 1:500, Invitrogen), detected by AlexaFluor488 and AlexaFluor633-conjugated secondary antibodies (1:800 and 1:500 respectively, Invitrogen, Carlsbad, CA) and counterstained with Hoechst 33342 (1:200, H1399, Invitrogen). Confocal z-stacks were recorded using a Leica TCS5 SPII laser scanning confocal microscope. ImageJ was used to create z-projections and merged images (Schneider et al., 2012).

### Transgene mapping

Linker-mediated PCR was performed using NlaIII, BfaI and DpnII linkers (Dupuy et al., 2005; Davison et al., 2007), with PCR products resolved by gel electrophoresis and directly sequenced. For *Et(REx2-cFos:Gal4ff)y237*, the integration was confirmed using PCR on genomic DNA from single embryos, primers x382GL 5-ttttggcttccaccttgaac, x382GR 5-tccagctcgcgaacaataat and x382TL 5-caagaatctctagttttctttcttgc yielding 143 bp for the wildtype genomic band and 219 bp in transgenic fish. In an outcross of *Et(REx2-cFos:Gal4ff)y237; Tg(UAS:Kaede)s1999t*, both bands were present in all Kaede positive embryos (*n* = 25), confirming the integration site.

### Morpholinos

For *rest* knockdown experiments we used a previously characterized splice-blocking morpholino (Gene Tools, Philomath, OR) against the intron-exon boundary of zebrafish *rest* exon 3 (Gates et al., 2010), injected at 5 ng per embryo using standard techniques (Eisen and Smith, 2008), with controls receiving 5 ng of the Gene Tools standard control morpholino. The control and *rest* morpholinos were injected into a similar number of embryos from each batch, so that the effect of *rest* knockdown could be quantified using sibling controls.

### Bioinformatics

Analysis of NRSE sequences was performed on zebrafish genome assembly Zv9, downloaded from NCBI, with code written in IDL. For analyzing the distance of each NRSE to neighboring sites, a less stringent NRSE definition was used allowing 2 mismatches to the consensus because there is evidence that multiple weak sites may contribute to silencing (Mortazavi et al., 2006). Sequence logo was generated using Web logo (Crooks et al., 2004).

### Statistical analysis

During the enhancer trap screens, we retained the first 50 F1 lines showing any Kaede reporter expression regardless of location or perceived “quality.” As some lines were lost before they could be scored in the F2 generation, and others turned out to have multiple integrations which were separated during breeding, we ultimately analyzed expression in 206 lines. Larval expression pattern frequencies were compared using Chi-squared analysis; for *post-hoc*, a Tukey-type contingency test for multiple comparisons was used [*q*_(0.05, ∞, 4)_ = 3.63] (Zar, 1996). Significant results are reported by *P* < 0.05 and *q*_(0.05, ∞,4)_ > 3.63. SPSS and Gnumeric were used for statistical analysis.

## Results

### NRSE motifs suppress non-neuronal expression in transient transgenics

**Figure 1:**
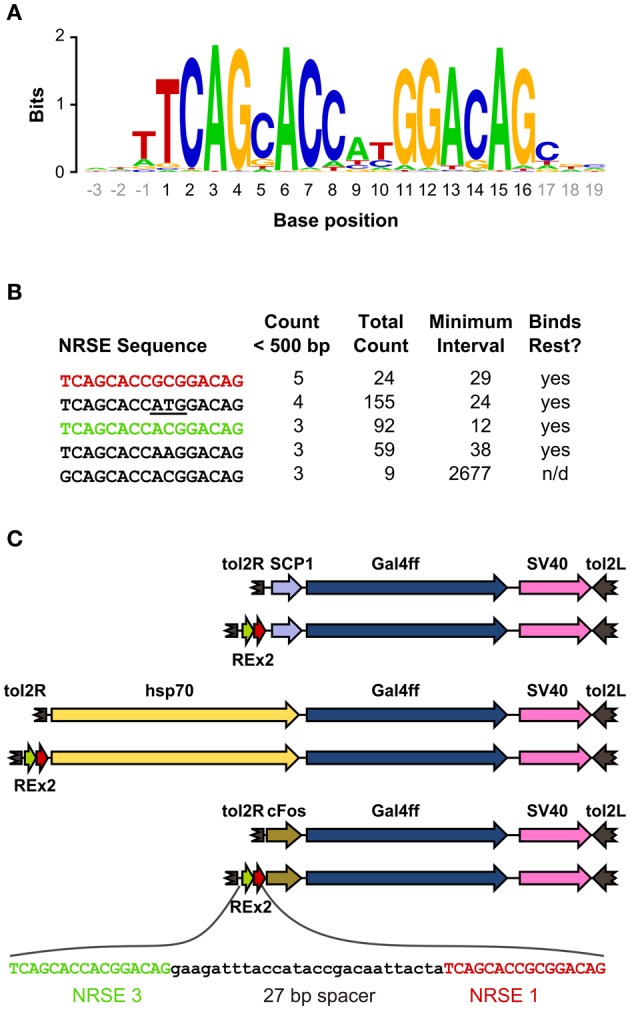
**Identification of NRSE sites for testing with basal promoters. (A)** Sequence logo representing the consensus zebrafish NRSE sequence. Base positions are relative to the core 16 bp element (black). Stack height at each position indicates the degree of conservation and letter height shows relative frequency. **(B)** Zebrafish NRSE sequences ranked by the number of instances where they are less than 500 bp from the transcription start site (Count <500 bp). Also shown for each: the total number of instances of each sequence in the genome, the minimum interval from a sequence to its neighboring NRSE and whether evidence has been reported for *rest* binding [taken from (Bruce et al., 2004), n/d, not determined]. The ATG in the second NRSE is underlined. **(C)** Schematic of the constructs tested, in order: SCP1:Gal4ff, REx2-SCP1:Gal4ff, hsp70:Gal4ff, REx2-hsp70:Gal4ff, cFos:Gal4ff, REx2-cFos:Gal4ff. The DNA sequence of the REx2 element, containing the third (green) and first (red) NRSE sequences from **(B)** is expanded. Elements are shown to scale, except that only part of the tol2 arms at either end of the construct are represented (broken brown arrows).

**Figure 2:**
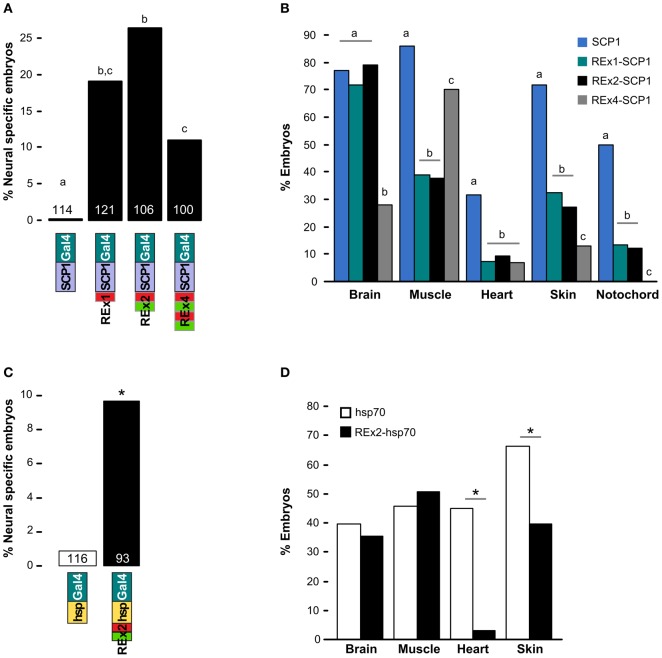
**Testing NRSE derived DNA elements for suppression of non-neuronal reporter expression. (A,B)** Transient expression of Gal4ff reporter constructs with different combinations of NRSE elements (green and red boxes) at the 5′ end of a SCP1 basal promoter. **(A)** Percentage of 48 hpf embryos where cells expressing the reporter were observed only in the nervous system. The number of embryos examined for each construct is indicated. Letters show *post-hoc* Tukey tests, *p* < 0.05 where letters differ. **(B)** Percentage of embryos with any cells observed in the indicated tissues after injection with SCP1:Gal4 (blue), REx1-SCP1:Gal4 (green), REx2-SCP1 (black) and REx4-SCP1 (gray) plasmids. *N* is for the cognate construct in **(A)**. Letters show *post-hoc* tests for multiple proportions for each tissue type, *p* < 0.05 where letters differ. **(C)** and **(D)** Transient expression of hsp70:Gal4ff (white bars) and REx2-hsp70:Gal4ff (black bars) plasmids. **(C)** Percentage of embryos with reporter expression only in brain or spinal cord and number of embryos examined for each construct. ^*^p < 0.005. **(D)** Percentage of embryos with any expression in the indicated tissues. ^*^p < 0.001.

We scanned the zebrafish genome with the core NRSE consensus motif TCAGCACCnnGGACAG (Bruce et al., 2004; Mortazavi et al., 2006), accepting one mismatch (Figure 1A). This identified 1102 potential NRSE sites, similar to the previously reported number of sites in zebrafish (Mortazavi et al., 2006), comprising 242 different sequences. As we intended to test NRSE sites by placing multiple elements upstream of a small core promoter, we ranked the 242 putative NRSE elements by the number of times they were found within 500 bp of a transcriptional start site and determined the minimum distance to the nearest neighbor NRSE (Figure 1B). Three of the top ranked elements were perfect matches to the consensus and occurred at least once within 30 bp of another NRSE element. These elements had all been previously demonstrated to bind Rest (Bruce et al., 2004) and were adjacent to genes known to be brain specific. All three were previously identified as among the most common NRSE elements in *Fugu rubripes* (Bruce et al., 2004). The second ranked sequence contains an ATG codon in a strong Kozak context (accATGg) which could give rise to spurious transcription in a transgene. We therefore tested the efficacy of the 1st and 3rd ranked NRSE motifs in suppressing non-neuronal expression in zebrafish. Evidence suggests that variants of the NRSE motif have different affinities for Rest and facilitate repression in different tissues (Bruce et al., 2004). We performed transient expression experiments, injecting *Tg(UAS:Kaede)s1999t* embryos (Davison et al., 2007) with DNA constructs in which a basal super core 1 (SCP1) promoter and Gal4ff reporter (Asakawa et al., 2008) were coupled to one of three different NRSE containing motifs: a single common NRSE element (REx1), two different NRSEs separated by a 27 bp spacer (REx2), and tandem copies of the REx2 element (REx4). NRSE motifs were placed immediately upstream of the promoter (Figure 1C). The SCP1 promoter had not been previously used in zebrafish, but was chosen because it combines strong versions of basal promoter elements to give very high level expression in mammalian cell culture (Juven-Gershon et al., 2006). Consistent with this, embryos injected with the SCP1:Gal4ff construct did not show expression only in brain (*n* = 0/114 injected embryos). In contrast, brain specific expression was observed in at least 10% of embryos injected with each of the three NRSE containing constructs [*X*^2^_(*df* = 3)_ = 35.3, *p* < 0.001] (Figure 2A).

The REx2 containing construct yielded the highest proportion of embryos in which only neuronal expression was observed (26.4%; REx2-SCP1 compared to SCP1, *q* = 12.76, *p* < 0.001) (Figure 2A). The REx4 element more strongly suppressed expression in skin and notochord but also suppressed expression in brain [*X*^2^_(*df* = 3)_ = 79.70, *p* < 0.001; percentage with brain in REx4-SCP1 compared to SCP1, *q* = 14.93, *p* < 0.001] (Figure 2B). Examination of expression in tissues known to produce severe morphological ablation phenotypes showed that the REx2 element strongly suppressed expression in heart [*X*^2^_(*df* = 3)_ = 39.35, *p* < 0.001; REx2-SCP1 compared to SCP1, *q* = 9.44, *p* < 0.001], notochord [*X*^2^_(*df* = 3)_ = 67.98, *p* < 0.001; *q* = 10.66, *p* < 0.001], skin [*X*^2^_(*df* = 3)_ = 89.83, *p* < 0.001; *q* = 13.59, *p* < 0.001] and muscle [*X*^2^_(*df* = 3)_ = 78.51, *p* < 0.001; *q* = 15.44, *p* < 0.001].

To confirm the efficacy of the REx2 element, we tested whether it could also suppress non-neuronal expression by the hsp70 promoter, which has previously been used for enhancer trapping in zebrafish (Feng et al., 2007; Scott et al., 2007). We inserted the REx2 element at the 5′ end of the promoter, in this case 767 bp from the translational start site (Figure 1C). The REx2 element increased brain-specific expression 11.2-fold [*X*^2^_(*df* = 1)_ = 8.81, *p* < 0.005] (Figure 2C), suppressing expression in heart [*X*^2^_(*df* = 1)_ = 46.07, *p* < 0.001] and skin [*X*^2^_(*df* = 1)_ = 14.72, *p* < 0.001], but not in muscle [*X*^2^_(*df* = 1)_ = 0.49, 0.75 > *p* > 0.50] or brain [*X*^2^_(*df* = 1)_ = 0.38, 0.75 > *p* > 0.50] (Figure 2D). Ectopic expression is commonly seen in transient transgenic assays. The reduction in non-neuronal expression with constructs including the REx2 element was therefore encouraging, but to definitively show its effectiveness we next sought to show that it suppresses expression outside the nervous system in stable transgenic lines.

### Efficacy of REx2 element for enhancing trapping

**Figure 3:**
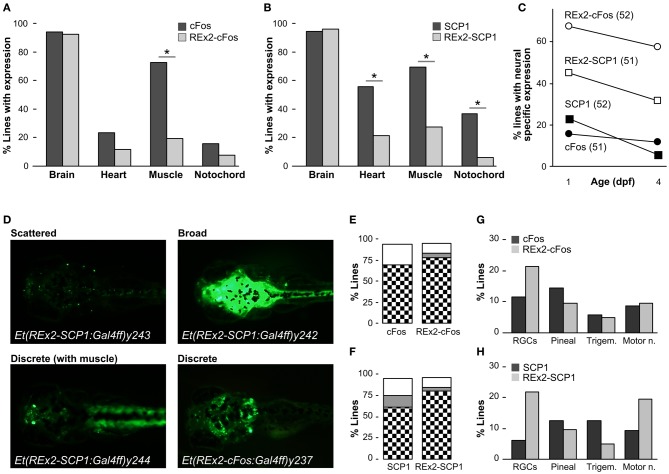
**Recovery of brain specific enhancer trap lines using the REx2 element. (A)** Percentage of enhancer trap lines recovered with expression in the indicated tissues at 4 dpf, using a cFos:Gal4ff construct (51 lines, dark bars) and a REx2-cFos:Gal4ff construct (52 lines, light bars). **(B)** As for **(A)**, but with enhancer trap constructs SCP1:Gal4ff (52 lines, dark bars) and REx2-SCP1:Gal4ff (51 lines, light bars). **(C)** Percentage of lines with expression only observed in the nervous system at 1 dpf and 4 dpf for each of the enhancer trap constructs used. **(D)** Examples of Gal4 enhancer trap lines with stochastic, discrete and broad expression in the brain. Lines were crossed to *Tg(UAS:Kaede)s1999t* for visualizing expression. **(E)** and **(F)** show the percentage of lines with expression in discrete brain structures (checks), broad expression through the brain (gray) or stochastic asymmetric expression in the brain (white) for the indicated enhancer trap constructs. **(G)** Percentage of lines with expression in the indicated neuronal cell types at 4 dpf for enhancer trap lines generated using the cFos:Gal4ff (dark bars) and REx2-cFos:Gal4ff (light bars) constructs. **(H)** As for **(G)** but with SCP1:Gal4ff (dark bars) and REx2-SCP1:Gal4ff (light bars). ^*^p < 0.001 for all panels.

To characterize whether the REx2 element also increased the recovery of brain specific enhancer trap lines, we performed two enhancer trap screens, comparing recovery of brain specific Gal4ff reporter transgenic lines with, and without the REx2 element (Figure 1C). For the first screen we used the cFos promoter, a well-characterized basal promoter which is silent in the absence of transcriptional activating enhancer elements (Fisher et al., 2006) and which has previously been used in a zebrafish enhancer trap (Scott and Baier, 2009). To visualize expression patterns we maintained all lines with *Tg(UAS:Kaede)s1999t*. Since most founder fish contained multiple transgene integrations resulting in overlapping reporter expression patterns (average 3.6), we bred each through multiple generations to isolate transgenic lines with unique expression patterns that segregated close to Mendelian ratios. For each enhancer trap construct we kept the first 50 lines showing any reporter expression irrespective of the pattern of expression in order to quantitatively compare the effectiveness of the REx2 element. We examined larvae at 1 dpf and 4 dpf for expression in brain, heart, muscle, or notochord. As in previous enhancer traps, almost all lines recovered showed some degree of expression in the brain. Lines that were made using the REx2 containing vector showed a marked reduction in expression outside the nervous system [*X*^2^_(*df* = 1)_ = 23.89, *p* < 0.001], that was highly significant for muscle [*X*^2^_(*df* = 1)_ = 30.96, *p* < 0.001] (Figure 3A). Recovery of brain specific lines increased 4.3-fold at 1 dpf (*q* = 10.64, *p* < 0.05) and 4.9-fold at 4 dpf (*q* = 9.86, *p* < 0.05) (Figure 3C).

For the second enhancer trap screen, we used the SCP1 promoter, speculating that although it is sufficient to drive strong expression alone, it might nevertheless be susceptible to regulation by genomic enhancers and silencers of transcription. Consistent with its strong basal promoter activity, the SCP1 promoter tended to show a higher level of activity in non-neuronal tissues than the cFos promoter in stable transgenics [*X*^2^_(*df* = 3)_ = 8.38, *p* < 0.05]. Again, lines containing the REx2 element had significantly reduced expression outside the nervous system [heart, *X*^2^_(*df* = 1)_ = 12.68, *p* < 0.001, muscle, *X*^2^_(*df* = 1)_ = 17.99, *p* < 0.001, notochord, *X*^2^_(*df* = 1)_ = 14.41, *p* < 0.001] (Figure 3B) such that recovery of brain specific lines increased 2.0-fold at 1 dpf (*q* = 4.69, *p* < 0.05) and 5.4-fold at 4 dpf (*q* = 6.92, *p* < 0.05) (Figure 3C).

Improvements in the proportion of brain specific lines would not be experimentally useful unless the lines recovered marked neuroanatomically distinct structures. For lines with any expression in the brain we classified 4 dpf neuronal expression patterns as “stochastic” (scattered asymmetric expression in the brain, varying between larvae in a clutch), “discrete” (reproducibly marking the same set of neuroanatomical structures) or “broad” (showing uniform expression throughout the brain) (Figure 3D). For the cFos promoter, inclusion of the REx2 element did not significantly alter the proportion of lines categorized as stochastic, discrete or broad [*X*^2^_(*df* = 3)_ = 6.14, 0.25 > *p* > 0.1] (Figure 3E). However, lines recovered from the REx2-SCP1 enhancer trap showed a small increase in the proportion of lines with neuroanatomically discrete expression patterns [*X*^2^_(*df* = 1)_ = 4.43, *p* < 0.05] (Figure 3F).

**Figure 4:**
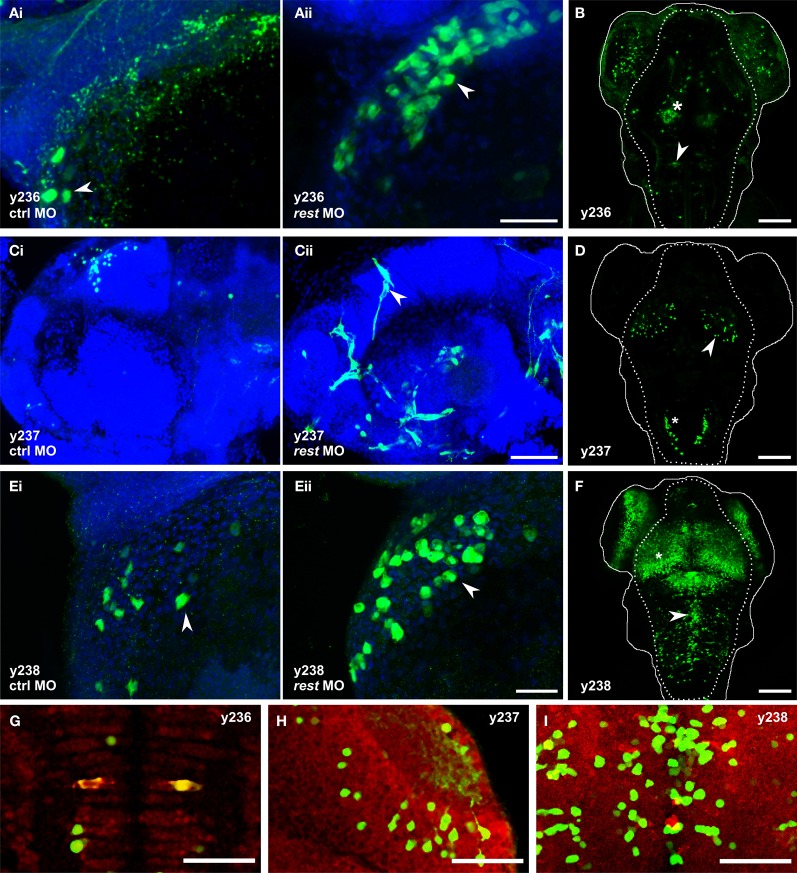
**Knockdown of *rest* reveals non-neuronal expression in REx2 enhancer trap lines.** In **(A,C,E)** confocal stacks show 2 dpf embryos immunostained with anti-Kaede (green), counterstained with Hoechst (blue). **(B,D,F)** are live confocal stacks of Kaede expression in 4 dpf embryos with the outline of the head (solid line) and brain (dotted line). **(A)** *Et(REx2-cFos:Gal4ff)y236;Tg(UAS:Kaede)s1999t* embryos injected with (i) control morpholino or (ii) morpholino targeting *rest*. Arrowheads show heart cells. **(B)** At 4 dpf *y236* larvae show reporter expression in hypothalamus (asterisk) and the Mauthner cells (arrowhead). **(C)** *Et(REx2-cFos:Gal4ff)y237;Tg(UAS:Kaede)s1999t* embryos injected with (i) control morpholino or (ii) morpholino targeting *rest*. Arrowheads show cranial vasculature. **(D)** At 4 dpf *y237* larvae show reporter expression in the optic tectum (arrowhead) and caudal hindbrain (asterisk). **(E)** *Et(REx2-cFos:Gal4ff)y238;Tg(UAS:Kaede)s1999t* embryos injected with (i) control morpholino or (ii) morpholino targeting *rest*. Arrowheads show heart cells. **(F)** At 4 dpf *y238* larvae show reporter expression in the optic tectum (asterisk) and a medially located population of cells in the hindbrain (arrowhead). **(G,H,I)** are single 2 μm confocal slices for 3 dpf embryos co-stained for Kaede expression (green) and the neuronal marker elav1 (red), centered on the regions marked with the arrowheads in **(B,D,F)** respectively. Scale bars: **(A,C,E)** 40 μm, **(B,D,F)** 100 μm, **(G,H,I)** 50 μm.

In both the REx2-cFos and REx2-SCP1 enhancer traps, 80% of lines recovered labeled a discrete population of cells in the nervous system. This would be misleading if the REx2 motif simply lead to repeated recovery of lines with a particular expression pattern. We scored four easily recognized neuronal cell groups and calculated the proportion of the discrete lines in which any of those groups were labeled (Figures 3G,H). Inclusion of the REx2 element did not significantly alter the recovery of lines with bias in a particular expression pattern for either cFos [*X*^2^_(*df* = 3)_ = 1.45, 0.75 > *p* > 0.5] or SCP1 [*X*^2^_(*df* = 3)_ = 5.0, 0.25 > *p* > 0.1]. As a final metric, we scored lines which had specific brain expression in a very restricted pattern, labeling only a small group of nuclei or cluster of cells (see for example *Et(REx2-cFos:Gal4ff)y236* in Figure 4D). Of the 16 lines that met this stringent standard, 15 were made using a construct containing the REx2 element [cFos, *X*^2^_(*df* = 1)_ = 5.53, *p* < 0.05; SCP1, *X*^2^_(*df* = 1)_ = 7.66, *p* < 0.01]. Together, these assessments demonstrate that the REx2 element leads to a substantial improvement in the recovery of enhancer traps which cleanly label specific groups of cells in the brain. Such lines are the most useful for mapping brain circuitry because they permit manipulations of small populations of cells within the brain.

### REx2 element suppress non-neuronal expression through rest

To test our working hypothesis that the REx2 element improves brain specific enhancer trapping by selectively suppressing non-neuronal expression of the transgene, we injected a morpholino against *rest* mRNA into lines which showed little or no expression outside the brain, reasoning that this should expand the pattern of reporter expression into transgenic lines where the REx2 element normally suppresses non-neuronal expression. We used a previously described splice-blocking morpholino which allows near normal embryonic development through 72 hpf (Gates et al., 2010). Using qPCR, we confirmed that as reported, at 5 ng per embryo, knockdown is robust through 24 hpf with an 85.5% reduction in *rest* expression (*t*-test, *p* < 0.001). Injection of the *rest* morpholino into cFos:Gal4 enhancer trap lines (lacking the REx2 element) revealed no changes in expression at days 1–3 when *rest* knockdown embryos show only minor morphological abnormalities (*n* = 5 lines). When injected into REx2-cFos:Gal4 lines, we saw changes in expression consistent with de-repression of the reporter in non-neuronal tissues in 3 of 10 lines injected.

In *Et(REx2-cFos:Gal4ff)y236;Tg(UAS:Kaede)s1999t* embryos, we saw Kaede expression in the hatching gland at 24 hpf in morphants (*n* = 22/27), but not in embryos injected with control morpholino [*n* = 0/28; *X*^2^_(*df* = 1)_ = 39.6, *p* < 0.001]. By 30 hpf, morphants showed expression in the heart, which strengthened through 48 hpf while control injected embryos showed little or no heart expression [number of heart cells expressing reporter: control 1.7 ± 0.8, morphants 5.0 ± 1.1, *t*-test *t*_(9.1)_ = 2.5, *p* = 0.036, *N* = 6 embryos each] (Figure 4A). By 4 dpf, *y236* larvae show strong expression in the hypothalamus, weaker expression in the olfactory bulb and only stochastic labeling of cells outside the brain (Figure 4B). In a second line, *Et(REx2-cFos:Gal4ff)y237; Tg(UAS:Kaede)s1999t*, uninjected and control morpholino injected embryos show only brain expression at the caudal edge of the otic vesicle at 30 hpf, with weak variegated expression of Kaede in intersegmental vessels in a minority of embryos at 48 hpf (*n* = 5/25). Injection of the *rest* morpholino into this line strengthened expression in trunk intersegmental vessels (*n* = 25/30) with additional cranial vasculature expression not seen in controls at 48 hpf (Figure 4C). From 48 hpf through 4 dpf, *y237* embryos mark a small population of neurons in the optic tectum and caudal hindbrain (Figure 4D). *Et(REx2-cFos:Gal4ff)y238; Tg(UAS:Kaede)s1999t* embryos have strong expression in the forebrain, ventral retina, pronephric ducts and caudal tail fin at 24 hpf. At 48 hpf, there is also weak expression in the heart. Injection of the *rest* morpholino led to a robust increase in heart expression [number of heart cells expressing reporter: control 3.2 ± 1.1, morphants 10.0 ± 2.2, *t*-test *t*_(6.3)_ = 2.7, *p* = 0.034, *n* = 5 embryos each] (Figure 4E). By 4 dpf, *y238* larvae have additional expression in the optic tectum, hindbrain and trunk neuromasts (Figure 4F).

**Figure 5:**
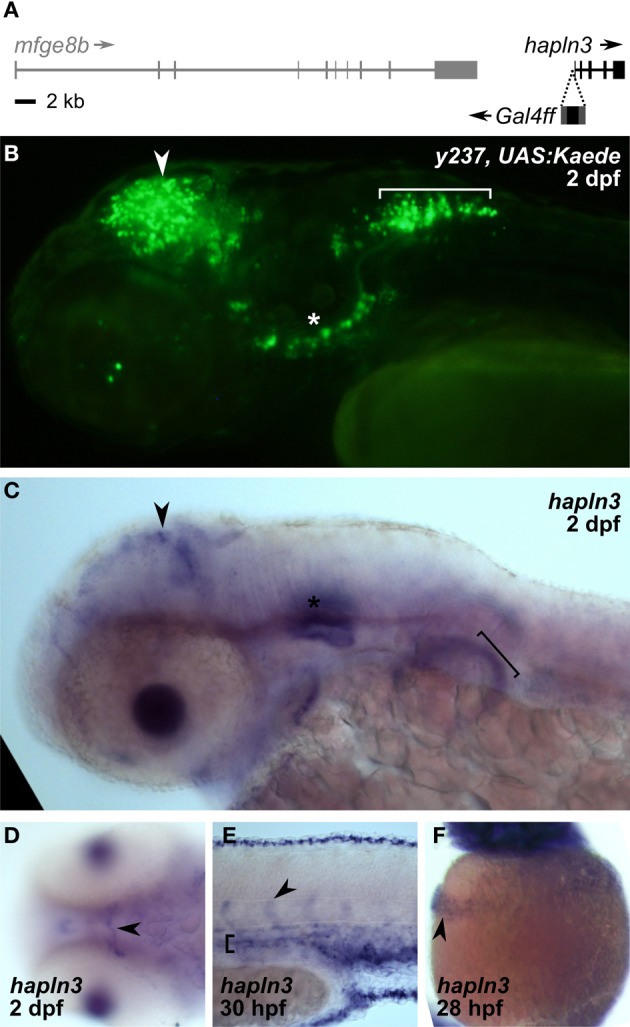
**REx2 enhancer trap *y237* recapitulates tectal expression of the adjacent *hapln3* gene. (A)** The Gal4ff reporter in *y237* is integrated on chromosome 3, between *mfge8b* and *hapln3*, 190 bp from the first exon of *hapln3*. **(B)** *y237* transgenic larvae show Kaede reporter expression in the optic tectum (arrowhead), adjacent to the otic vesicle (asterisk) and in the hindbrain (bracket). Lateral view, 2 dpf. **(C)** WISH (2 dpf) shows that *hapln3* is expressed in the optic tectum (arrowhead), in the otic vesicle (asterisk) and non-neuronal tissues including pectoral fin (bracket). **(D)** Dorsal view of head (2 dpf) reveals cranial vasculature expression of *hapln3*, arrowhead shows basal communicating artery (Isogai et al., 2001). **(E)** Lateral view of tail (30 hpf) shows expression of *hapln3* in the dorsal aorta (bracket), the source of cells which form intersegmental vessels (arrowhead). **(F)** Lateral view of yolk (28 hpf) showing *hapln3* in hatching gland (arrowhead).

We asked whether the expanded expression patterns seen with *rest* morpholino injection specifically reflects recovery of the normal non-neuronal pattern of expression of genes adjacent to the integration site whose enhancers are driving expression of the reporter. We were able to map the integration site for two lines. *Et(REx2-cFos:Gal4ff)y238* integrated at chr3:10432994. Since the nearest annotated genes, *rnps1* and *cbx2* both show broad patterns of expression (Thisse and Thisse, 2004), it is possible that the heart expression after *rest* morpholino injection reflects recovery of the non-neuronal expression domain of one of these genes. *Et(REx2-cFos:Gal4ff)y237* mapped to chr25:19691130 (zv9), within the promoter region of *hyaluronan and proteoglycan link protein 3 (hapln3)*, 190 bp from the start of transcription (Figure 5A). Reporter expression in the brain of *y237* transgenic larvae resembles that of *hapln3* with both showing expression in the optic tectum and adjacent to the otic vesicle (Figures 5B,C). Transgenic larvae show an additional locus of expression in the hindbrain not detected by *in situ* hybridization for *hapln3*. However, *hapln3* is expressed in several areas outside the nervous system, including in the hatching gland, pectoral fin, median fin fold, and vasculature, including cranial and trunk vasculature (Thisse and Thisse, 2004) (Figures 5C–F). This pattern of non-neuronal expression is not observed in *y237* larvae, which could be due to either incomplete regulatory control of the transgene, or due to the REx2 motif suppressing non-neuronal expression. The vasculature expression domain is partially restored in *rest* morphant *y237* larvae, suggesting that that the REx2 element suppresses non-neuronal expression of the reporter that would normally be driven by local genomic enhancers.

## Discussion

Here we define a short DNA fragment containing two NRSE sequences that suppresses expression outside the nervous system. Guided by a ChIP analysis that showed greatest occupancy of NRSE sites in genes containing two different sites within 30 bp of each other (Bruce et al., 2004), we designed the REx2 element using zebrafish NRSE elements that (1) appear within 500 bases of a transcriptional start site, (2) are situated within 30 bp of another NRSE element and (3) were previously shown to bind Rest. Our data provides quantitative evidence that when included in an enhancer trap vector, this element strongly enriches the recovery of transgenic lines with expression limited to the nervous system. REx2 transgenics robustly label diverse neuronal subtypes and yield high quality expression patterns. This method is thus a simple and effective way to restrict transgene expression to the nervous system.

We considered alternative strategies for confining enhancer trap expression to the brain. Based on our transient transgenic data, we expected that the REx2 element would also suppress expression of a reporter gene from a UAS-E1b promoter. This was not the case, nor were we able to suppress expression from the UAS-E1b promoter when the REx2 motif was placed in an intron downstream of this promoter (data not shown). Previous work has shown that Gal4 can reactivate silenced UAS-E1b transgenic reporters (Goll et al., 2009) and it is possible that Gal4 similarly reverses Rest mediated transcriptional silencing. However, as many zebrafish Gal4 lines with expression both in the brain and non-neuronal tissues exist, we are continuing work to extend this technique to enable suppression of non-neuronal expression from UAS reporter lines.

An intersectional method could also be used to restrict enhancer trap expression to the nervous system, for instance by breeding Gal4 lines with double transgenic *HuC:Cre; UAS:lox-STOP-lox-nfsB-mCherry* fish. In this case, brain specific expression of Cre by the HuC promoter (Park et al., 2000) would ensure that Gal4 only activates NTR-mCherry expression in the nervous system. Intersectional strategies have proven highly successful in *Drosophila*, and are feasible in zebrafish (Faucherre and Lopez-Schier, 2011; Fujimoto et al., 2011). We did not pursue this approach because of the additional generation of breeding required to obtain triple transgenic fish and the low proportion of triple transgenic embryos expected from crosses. Both factors would tend to reduce the throughput of a large scale circuit breaking screen.

The REx2 element suppressed non-neuronal expression not only from the short cFos and SCP1 basal promoters, but also reduced expression in heart and skin when upstream of a 640 bp fragment of the hsp70 promoter. It may therefore also aid in refining expression to the nervous system in transgenic lines using defined promoter elements. As the element was not effective with the UAS-E1b promoter and did not suppress muscle expression from the hsp70 promoter, efficacy is likely to vary with different promoters. We note that in mammals, *Rest* is expressed in immature neurons (Ballas et al., 2005) and at low levels in adult hippocampal neurons (Sun et al., 2005). Although the region homologous to hippocampus is small or absent in zebrafish larvae, *rest* is expressed prior to neuronal differentiation in the zebrafish CNS (Gates et al., 2010; Wang et al., 2012) and it will therefore be interesting to see whether transgenic lines made with the REx2 motif show reduced expression in neuronal precursor cells. We recognized distinctive mature neuronal cell types in REx2 containing enhancer trap lines including retinal ganglion cells, trigeminal sensory neurons, motor neurons, and reticulospinal neurons. Moreover co-staining for the neuronal marker elav1 confirmed that many cells in *y236*, *y237*, and *y238* enhancer trap lines are neurons (Figures 4G–I). Thus, the REx2 enhancer trap lines do indeed label neurons.

By morpholino knockdown of *rest* mRNA, we saw increased expression of REx2 containing transgenes in non-neuronal tissues. Our data is therefore consistent with the REx2 element suppressing expression that would normally be activated by enhancer elements which direct expression outside the nervous system. Previously it has been demonstrated that an NRSE confers brain specific expression in transgenic *Xenopus* (Tan et al., 2010) and in zebrafish, a recent report has shown de-repression of non-neuronal reporter expression in NRSE containing enhancer trap lines in a *rest* mutant background (Kok et al., 2012). The partial recovery of non-neuronal expression patterns in *rest* morphant transgenic larvae likely reflects incomplete morpholino knockdown of *rest* transcript and non-neuronal expression is expected to be more extensive in the *rest* mutant background (Kok et al., 2012).

Existing enhancer trap reporter lines show significant levels of expression outside the nervous system. These lines have proven invaluable for functional neuroanatomical studies where reporter function can be experimentally triggered or monitored in a localized manner (Del Bene et al., 2010; Lee et al., 2010; Schoonheim et al., 2010) however, morphological defects after cell death in non-neuronal tissues make most of these lines unsuitable for an ablation based circuit breaking screen. Our preliminary data from ablating cells in Gal4 enhancer trap lines using a UAS:NfsB-mCherry reporter indicates that around half of the SCP1 lines tested die or show severe morphological defects after ablation, while only 10% of the REx2-SCP1 lines have similar phenotypes.

Transgenic lines in which reporter expression is confined to the nervous system are also essential for other circuit analysis techniques, particularly in unrestrained animals where reporter function needs to be localized genetically. Channelrhodopsin2 (ChR2) can be used for controlling neuronal activity in freely swimming zebrafish (Zhu et al., 2009), but ChR2 is also a robust modulator of muscle cell contraction (Arrenberg et al., 2010). Tracking neuronal activity in free swimming larvae using bioluminescent calcium imaging is a powerful method (Naumann et al., 2010), but with the confound that muscle calcium transients are large and likely to swamp signals from neurons during swim bouts (Cheung et al., 2011). The advance we report here will thus enhance the application of powerful circuit mapping techniques for analyzing the neuronal basis of behavior in zebrafish.

### Conflict of interest statement

The authors declare that the research was conducted in the absence of any commercial or financial relationships that could be construed as a potential conflict of interest.
